# Evaluating the benefits of machine learning for diagnosing deep vein thrombosis compared with gold standard ultrasound: a feasibility study

**DOI:** 10.3399/BJGPO.2024.0057

**Published:** 2024-12-18

**Authors:** Kerstin Nothnagel, Mohammed Farid Aslam

**Affiliations:** 1 Population Health Sciences, Canynge Hall, Bristol Medical School, University of Bristol, Bristol, UK; 2 Imperial College London, London, England, UK

**Keywords:** clinical (general), screening, diagnosis, venous thrombosis, artificial intelligence

## Abstract

**Background:**

This study evaluates the feasibility of remote deep venous thrombosis (DVT) diagnosis via ultrasound sequences facilitated by ThinkSono Guidance, an artificial intelligence (AI) app for point-of-care ultrasound (POCUS).

**Aim:**

To assess the effectiveness of AI-guided POCUS conducted by non-specialists in capturing valid ultrasound images for remote diagnosis of DVT.

**Design & setting:**

Over a 3.5-month period, patients with suspected DVT underwent AI-guided POCUS conducted by non-specialists using a handheld ultrasound probe connected to the app. These ultrasound sequences were uploaded to a cloud dashboard for remote specialist review. Additionally, participants received formal DVT scans.

**Method:**

Patients underwent AI-guided POCUS using handheld probes connected to the AI app, followed by formal DVT scans. Ultrasound sequences acquired during the AI-guided scan were uploaded to a cloud dashboard for remote specialist review, where image quality was assessed, and diagnoses were provided.

**Results:**

Among 91 predominantly older female participants, 18% of scans were incomplete. Of the rest, 91% had sufficient quality, with 64% categorised by remote clinicians as 'compressible' or 'incompressible'. Sensitivity and specificity for adequately imaged scans were 100% and 91%, respectively. Notably, 53% were low risk, potentially obviating formal scans.

**Conclusion:**

ThinkSono Guidance effectively directed non-specialists, streamlining DVT diagnosis and treatment. It may reduce the need for formal scans, particularly with negative findings, and extend diagnostic capabilities to primary care. The study highlights AI-assisted POCUS potential in improving DVT assessment.

## How this fits in

As the global population continues to age, the prevalence of deep venous thrombosis (DVT) is on the rise. Presently, DVT diagnostic protocols necessitate specialised equipment operated by trained personnel. The advent of artifical intelligence (AI)-guided point-of-care ultrasound (POCUS) offers the potential for non-specialists to conduct DVT scans. If proven accurate and feasible, the implementation of AI-guided POCUS could alleviate pressure on secondary care services, enhance DVT diagnostic availability in primary care, and contribute to greater efficiency within the NHS, ultimately reducing diagnostic expenses.

## Introduction

The National Institute for Health and Care Excellence (NICE) reports that deep vein thrombosis (DVT) has an annual incidence of 1–2 per 1000 people.^1^ Ensuring an accurate and prompt diagnosis is vital to forestall complications such as pulmonary embolism (PE) or post-thrombotic syndrome (PTS).^2^ Although progress has been made, DVT diagnosis is embedded in a complex clinical pathway incorporating risk assessment, laboratory analyses, and ultrasound, the latter typically performed in secondary care. The Wells score categorises patients into low, intermediate, and high risk, with a subsequent D-dimer blood test often used to rule out DVT in patients who are intermediate risk, despite its low specificity.^3^ Nevertheless, the traditional approach involves utilising compression ultrasound in conjunction with Doppler ultrasound (duplex). This method involves observing intraluminal contents and increases accuracy by revealing potential lack of blood flow where thrombosis is present but not visible.^4^

Previous studies have demonstrated that POCUS for DVT diagnosis, even without AI support, exhibits high sensitivity and specificity.^5^ For example, this has been shown in the meta-analysis conducted by Lee e*t al,* ^5^ in the randomised controlled trial by Bernardi *et al,* ^6^ and the systematic review and meta-analysis by Kraaijpoel *et al* ^7^, which concluded that POCUS of the proximal leg yields results comparable with standard DVT diagnostic ultrasound.

The need for more accessible diagnostic methods is evident, and the integration of AI-guided POCUS in primary care holds promise to address these challenges. Ultrasound effectiveness remains contingent on user proficiency. Although studies suggest that less trained healthcare professionals (HCPs) can reliably perform POCUS examinations,^4^ specific training requirements remain unclear.^8^ Consequently, highly trained specialists, including radiologists, and ultrasound technicians, are often required for accurate documentation,^9^ and liability mitigation, incurring significant costs, and longer waiting times.^10^ AI-guided POCUS has the potential to reduce the need for secondary care admissions and enhance access to DVT diagnostics in primary care settings. Particularly, it would benefit the most vulnerable patient groups such as the housebound, frail, and multimorbid. The implementation of this technology aligns with the goals outlined in the NHS England's long-term plan, which envisions to improve access to diagnostics in primary care through use of innovative technologies.^11^ The aim of this feasibility study was to:

1. Evaluate AI-guided POCUS effectiveness in guiding HCPs without formal ultrasound training to obtain ultrasound sequences of sufficient quality for remote interpretation.

2. Investigate if remote specialists can accurately provide a diagnosis based on AI-guided POCUS acquired sequences, comparing their assessments with those from the reference scan.

3. Assess the efficiency as a triage tool, stratifying scans into high and low risk and potentially eliminating the need for additional scans by a specialist, thereby streamlining the diagnostic process.

## Method

### Patient recruitment

Patients with suspected DVT requiring an ultrasound scan (USS) were recruited consecutively at a hospital in Berlin, Germany, over a 3.5-month period in 2022. Forty-six per cent of the participants were inpatients, 2% had directly scheduled scan appointments, and the majority (52%) were referred from the primary care sector.

A statistical dichotomous endpoint power calculation was conducted by an Imperial College London statistician. The calculation was based on the understanding that approximately 15% of patients with symptoms indicative of DVT would test positive. To achieve a statistical power of 80%, a minimum of seven patients with positive DVT cases were needed to be included in the study.

Patients were eligible for inclusion if they were aged ≥18 years, demonstrated the capacity to consent, exhibited symptoms suggestive of DVT, and the DVT diagnostic algorithm indicated the necessity of a diagnostic USS.^12^

Each consenting patient underwent two distinct USS:

- index: AI-guided POCUS conducted by HCPs without formal DVT diagnostic ultrasound training; and
- reference: the second scan, a formal duplex scan, which was conducted by a specialist blinded to the index scan results.

### Index test

The AI-guided scans were conducted using ThinkSono Guidance, an app developed by the company ThinkSono GmbH. This app, installed on a smartphone, was connected to a Clarius L7 HD3 linear handheld ultrasound probe. The app had, at the time of the study, a Class 1 CE certificate (Supplementary Information S1) and was used under its intended purpose (Supplementary Information S2).

Prior validation of the ThinkSono Guidance has been documented in previous studies.^6,7^ The app guides users through a two-region POCUS, directing compressions in the groin areas and in the knee pit. If the procedure identifies insufficient compression, improper vessel alignment in the image, or probe displacement during compression, the application suggests redoing the compression. Users retain the choice to override these suggestions. After successfully gathering all compression points, sequences of compression (lasting 10 seconds each) are uploaded to a cloud-based dashboard for remote assessment.

**Figure 1.**
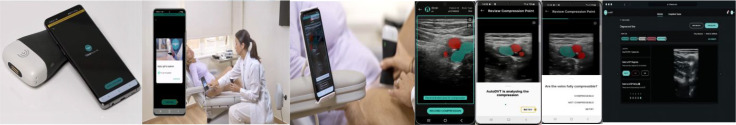
**A–G** Workflow of ThinkSono Guidance (it guides the operator through anatomical landmarks and indicates when to compress. ThinkSono Guidance analyses the images, and provides a remote diagnosis)

Four sequences are recorded per scan (Figure 1a–g). USS were conducted by a non-specialist without formal experience in performing DVT diagnostic scans, following a 1-hour training session with ThinkSono Guidance.

### AI algorithm

The ThinkSono Guidance app utilises a convolutional neural network for vessel analysis, supplemented by auxiliary branches for anatomical location prediction. Trained on prospectively collected data, the model employs manual vein and artery delineations along with image-level labels for anatomical locations. It operates on B-mode ultrasound images resampled to 128 × 128 pixels, producing segmentation masks and categorical location labels within 25 milliseconds.

The U-Net architecture serves as the model backbone, augmented by a landmark prediction branch. Three models, each tailored to specific anatomical regions, are trained with identical architecture. Further details and illustrations are provided in the figures and tables within the article.

The U-Net architecture serves as the model backbone, augmented by a landmark prediction branch. Three models, each tailored to specific anatomical regions, are trained with identical architecture. Further details and illustrations are provided in the attached figures.

### Reference standard

The DVT diagnostic scan, recommended by NICE guidelines, includes compressions at multiple points on the proximal leg, coupled with duplex.^12^ Compressions are typically applied every few centimetres from the groin to the knee pit along the large veins. Duplex is employed on the large veins to demonstrate normal blood movement. A qualified physician, unaware of the index scan outcomes, provided a report after completion of the reference scans, indicating the presence or absence of DVT.

### Image review

The ultrasound sequences from the index scan were assessed by five qualified remote experts using the secure cloud dashboard. These experts were kept unaware of all patient attributes and indications, only being informed if the examination was conducted on the left or right extremity.

Remote specialists assessed image quality using the American College of Emergency Physicians (ACEP) score, which is rated on a scale from 1–5. An overall examination score of ≥3 was deemed to indicate sufficient image quality (Table 1). Furthermore, each image scoring ≥3 was categorised as having 'compressible' veins, 'incompressible' veins, or requiring another USS ('indeterminate').

**Table 1.** American College of Emergency Physicians (ACEP) score

ACEP score	
**1**	No recognisable structures, no objective data can be gathered
**2**	Minimally recognisable structures, but insufficient for diagnosis
**3**	Minimal criteria met for diagnosis, recognisable structures but with some technical or other flaws
**4**	Minimal criteria met for diagnosis, all structures images well and diagnosis supported easily
**5**	Minimal criteria met for diagnosis, all structures images with excellent image quality and diagnosis completely supported

### Patient triaging

To mimic clinical triage methods, a retrospective analysis combined the classifications into high-risk and low-risk groups. This process aimed to reflect the proportion of scans that could potentially be managed in primary care without the need for referral to a formal USS. The scans were evaluated for image quality and compressibility assessment, as summarised in Table 2.

**Table 2.** Remote specialist reported diagnosis, index scan and sonographer diagnosis, and reference scan

	Reference scan positive	Reference scan negative
**Index scan positive**	9% TP	9% FP
**Index scan negative**	0% FN	83% TN

FN = false negative. FP = false positive. TN = true negative. TP = true positive.

### Statistical analysis

This study sought to assess the feasibility of utilising AI-guided POCUS to guide healthcare professionals (HCPs) without formal ultrasound training in acquiring ultrasound images suitable for remote interpretation. The evaluation included an analysis of image quality, the accuracy of remote diagnoses, and the efficiency of the AI-guided POCUS application as a primary care triage tool by categorising scans into high- and low-risk groups, potentially reducing the necessity for scans in secondary care.

For the reported confidence intervals (95% CI), statistical methods recommended for diagnostic test accuracy analysis were applied. This approach follows standard practices used in previous studies, including the development phase of ThinkSono Guidance (formerly AutoDVT), as outlined in the study by Prof. Kainz^13^. These methods were used to calculate the sensitivity and specificity of the AI-guided POCUS, contributing to the overall assessment of its diagnostic performance.

The analysis was conducted using Google Sheets, and the outcomes were displayed with variability divergence. The image quality assessment employed a majority voting process with five experts assigning an ACEP score of ≥3 for the uploaded scans. Remote diagnoses relied on the unanimous agreement of at least three experts regarding vein compressibility, and the results were compared with formal USS, considering sensitivity and specificity. The triaging into low- and high-risk categories was performed through majority voting by the remote specialists.

For the reported confidence intervals (95% CI), statistical methods recommended for diagnostic test accuracy analysis were applied. This approach follows standard practices used in previous studies, including the development phase of ThinkSono Guidance (formerly AutoDVT)^13^. These methods were used to calculate the sensitivity and specificity of the AI-guided POCUS, contributing to the overall assessment of its diagnostic performance.

## Results

### Participants

The 91 participants included 59% females, with an average age of 69.7 years. The mean body mass index (BMI) was calculated at 26, with the highest BMI recorded at 47.3 and the lowest at 15.8. It is noteworthy that only 59% of the 91 participants were referred for a scan based on a genuine suspicion of DVT. The remaining 41% of scans were conducted as exclusion diagnostics before initiating treatment for conditions such as cellulitis with antibiotics or for musculoskeletal issues requiring physiotherapy. This highlights that a majority of diagnostics, especially in secondary care, are performed as exclusion diagnostics.

**Figure 2.**
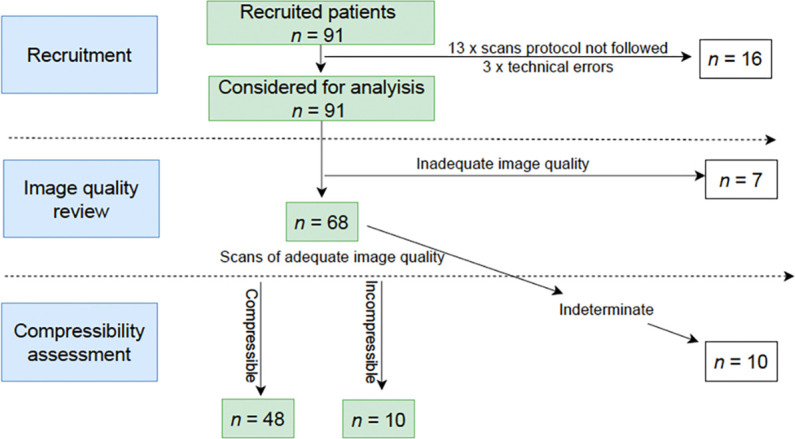
Flowchart

Thirteen of the 91 scans deviated from the study protocol, and an additional three encountered technical errors. These USS (n = 16, 18%) were either fragmented or not correctly transferred to the cloud dashboard, preventing an overall diagnosis (Figure 2). Consequently, these scans were categorised as high-risk group. Out of the 75 uploaded scans, 91% achieved an ACEP score of ≥3, indicating sufficient image quality for remote diagnosis as highlighted in Table 2.

However, in 15% of these scans, despite adequate image quality, a remote diagnosis could not be provided owing to issues such as other pathological findings, for example, a Baker's cyst or disagreement between the reviewers.

### Image quality

In the process of collective decision making, the five remote specialists allocated an ACEP score of ≥3 for 68 out of the 75 examinations, while seven examinations received an ACEP score <3. The average ACEP score for these exams was 3.35. Consequently, 75% (68/91) of the scans demonstrated adequate quality for diagnosis by remote clinicians (Table 4).

### Remote diagnosis

Table 2 Of the ultrasound scans reviewed by remote clinicians, 64% were categorised as either 'compressible' or 'incompressible,' aligning with the primary study objective of evaluating diagnostic feasibility (Table 4). However, 15% of the 68 scans encountered challenges that impeded a thorough assessment, including findings of other pathologies or disagreements among reviewers. Among the remaining 58 scans, the outcomes were as follows: 9% true positive (TP), 9% false positive (FP), 83% true negative (TN), and 0% false negative (FN), as illustrated in Table 3. This resulted in a sensitivity of 100% (95% confidence interval [CI] = 99.12% to 100%), indicating its ability to correctly identify all true positive cases, and a specificity of 90.57% (95% CI = 90.48% to 91.66%), highlighting its capability to accurately rule out the presence of DVT in the majority of cases. These findings suggest a high level of reliability in both sensitivity and specificity for AI-guided POCUS in diagnosing DVT.

### Risk triaging

If AI-guided USS with remote diagnosis would be employed as triage, 48% of 91 USS included in the study would have been categorised as high risk: 18% of 91 scans deviated from the study protocol, 8% had an ACEP score <3. Additionally, 11% were classified as needing to be repeated ('indeterminate') despite sufficient image quality and 11% of scans were categorised as 'incompressible'. In contrast, the remaining 53% of scans were marked as 'low risk', as each sequence displayed compressible veins with adequate image quality. In clinical settings, patients at low risk would potentially not require a formal USS (Table 3).

**Table 3.** Summary of key information

All scans	Result
Image quality^a^	75% (68/91) scans with ACEP ≥3
Remote diagnosis	64% (58/91) scans included for diagnosis
Risk triaging^b^	53% (48/91) of participants at low risk
**Scans considered for remote analysis**	
Image quality^a^	91% (68/75) scans with ACEP ≥3
Remote diagnosis	77% (58/75) scans included for diagnosis
Risk triaging^b^	64% (48/75) of participants at low risk

^a^Demonstrating adequate quality for remote interpretation. ^b^Suggesting potential avoidance of a formal scan in secondary care

**Table 4.** Risk triaging

Risk category	Criteria for designation
**High risk**	Incomplete examination
	At least 1 sequence with ACEP <3
	Incompressible veins
	Other reasons deemed by reviewer, for example, considerations for differential diagnosis such as a Baker's Cyst
**Low risk**	Complete examination
	All cine-loop sequences with ACEP ≥3
	All veins are identified as compressible

ACEP = American College of Emergency Physicians

## Discussion

### Summary

This study suggests that AI-assisted DVT diagnosis conducted by HCPs without formal ultrasound training can effectively capture quality images of proximal leg veins using AI-guided POCUS. This method shows promise for remote diagnosis and integration into primary care, potentially reducing the demand for formal ultrasounds, thereby enhancing diagnostic efficiency and resulting in cost savings.

### Strengths and limitations

While our study demonstrates promising outcomes, it has limitations. The statistical analysis was retrospective, and further prospective research is needed to validate the method’s safety and effectiveness. Additionally, the study had a small number of positive DVT cases, indicating the necessity for validation with larger cohorts. Moreover, logistical constraints prevented real-time feedback, potentially affecting the ratio of avoided formal ultrasounds.

### Comparison with existing literature

Our findings align with previous research on the reliability of POCUS for DVT diagnosis.^6,7^ Integrating AI technology has the potential to enhance POCUS accuracy further. However, there are concerns regarding documentation and liability when scans are conducted by non-experts. Our method addresses some of these concerns by integrating remote expert review and recommending a formal scan if suspicion for DVT remains high..

### Implications for research and practice

Integrating AI-guided DVT diagnosis into primary care could revolutionise diagnostic processes and extend diagnostic capabilities to underserved populations.^6^ This technology could streamline diagnostic pathways, particularly benefiting housebound or nursing home residents. Further research is needed to validate our findings in larger, multi-centre studies and to assess the generalisability of our results to broader healthcare contexts.

In conclusion, our findings support the feasibility of AI-guided POCUS with remote expert diagnosis. It could potentially offer a streamlined and cost-effective approach with the potential for improved patient outcomes.

## Trial registration number

ISRCTN24293748

## Data

The dataset relied on in this article is available from the corresponding author on reasonable request.
